# Ocular Therapeutics for Physicians and Students

**Published:** 1899-06

**Authors:** 


Ocular Therapeutics for Physicians and Students. By F. W. Max Ohlemann, M. D., (Minden, Germany,) Late Assistant Physician in the Ophthalmological Clinical Institute of the Royal Prussian University of Berlin, etc.; translated and edited by Charles A. Oliver, A. M., M. D. (Univ. Pa.), one of the Attending Surgeons to the Wills' Eye Hospital; one of the Ophthalmic Surgeons to the Philadelphia Hospital, etc. Two hundred and seventy-four pages. Philadelphia: P. Blakiston's Son & Co. 1899.

This is not designed as a text-book, in any sense, but is intended to be a collection of "the most important and the most useful formula" in ocular therapeutics. It is rather an elementary treatise, treating of massage, heat and cold, bandages, electricity, subconjunctival injections, astringents, antiseptics, ointments, mydriatics, myotics, local and general anesthesia, and remedies and formulæ applicable to special diseases of the eye. There is nothing particularly new in the book, but it will prove instructive and useful to the beginner in ophthalmology. A. A. H.

